# Clinical outcomes in hospitalized patients with community-acquired pneumonia: A comprehensive analysis of associated factors

**DOI:** 10.1371/journal.pone.0344171

**Published:** 2026-03-05

**Authors:** Deema Rahme, Hania Nakkash Chmaisse, Pascale Salameh

**Affiliations:** 1 Faculty of Pharmacy, Beirut Arab University, Beirut, Lebanon; 2 INSPECT-LB (Institut National de Santé Publique, d’Épidémiologie Clinique et de Toxicologie-Liban), Beirut, Lebanon; 3 Faculty of Pharmacy, Lebanese University, Hadat, Lebanon; 4 Gilbert and Rose-Marie Chagoury School of Medicine, Lebanese American University, Byblos, Lebanon; Aga Khan University, PAKISTAN

## Abstract

**Background:**

Community-acquired pneumonia (CAP) remains a significant cause of hospitalization and mortality globally. Optimizing clinical outcomes in CAP depends heavily on timely, appropriate empiric antibiotic therapy. However, limited data from low- and middle-income countries hinder effective stewardship efforts. The study aims to assess clinical outcomes among hospitalized CAP patients in Lebanon and identify key factors associated with deterioration or death, with particular emphasis on the role of guideline-concordant empiric antibiotic prescribing.

**Methods:**

A cross-sectional study was conducted in five tertiary hospitals across Lebanon between January and June 2024. Adult patients admitted with CAP were included. Demographic, clinical, laboratory, microbiological, and treatment data were extracted. Antibiotic regimens were evaluated for adherence to national CAP guidelines. Multivariable logistic regression was used to identify predictors of poor clinical outcomes, defined as ICU admission or in-hospital death.

**Results:**

Inappropriate antibiotic selection (aOR=18.81, p<0.001) and dosing (aOR=1.78, p=0.027) were significantly associated with poor outcomes. Additional predictors included advanced age, congestive heart failure, coronary artery disease, elevated WBC, and CURB-65 scores ≥3. Conversely, patients with classical CAP presentations (e.g., wheezing, rales) were more likely to experience favorable outcomes.

**Conclusion:**

Inappropriate empiric antibiotic prescribing significantly worsens clinical outcomes in hospitalized CAP patients. These findings underscore the urgent need for strengthening antimicrobial stewardship programs, implementing clinical decision support tools, and reinforcing physician education to promote adherence to national guidelines and improve patient safety.

## 1. Introduction

Community−acquired pneumonia (CAP) remains a major infectious disease challenge, contributing substantially to morbidity, mortality, and healthcare costs worldwide [1,2]. Within the Middle East and North Africa region, lower respiratory tract infections constitute a major contributor to disease burden, accounting for approximately one-tenth of all mortality and representing a public health priority [3,4].

Patient demographics, particularly advanced age, alongside the presence of comorbidities such as cardiovascular disease, diabetes mellitus, chronic obstructive pulmonary disease, and immunosuppressive states, are critical determinants of outcomes [5].

Clinical presentation features, including abnormalities in vital signs, evidence of respiratory distress, and altered mental status, function as key prognostic indicators that guide risk stratification and inform decisions regarding treatment intensity [6]. Furthermore, laboratory parameters including inflammatory biomarkers, renal function indices, and oxygenation metrics provide quantifiable measures of disease severity and physiological derangement that correlate with adverse outcomes [7,8].

Appropriate empirical antimicrobial therapy is a key modifiable determinant of CAP outcomes. Guideline-concordant treatment has been associated with reduced mortality and shorter hospitalization, whereas inappropriate initial therapy may contribute to treatment failure and adverse clinical outcomes [9–12].

The implications of antibiotic prescribing practices extend beyond individual patient outcomes to encompass broader public health concerns. Injudicious use of antimicrobial agents, particularly the overreliance on broad-spectrum antibiotics in the absence of microbiological justification, accelerates the emergence and dissemination of antimicrobial resistance (AMR). Healthcare facilities serve as critical centers for resistance development and transmission, where high antimicrobial selection pressure, vulnerable patient populations, and opportunities for pathogen spread converge [13]. The AMR crisis disproportionately affects resource-limited settings, where surveillance infrastructure, laboratory diagnostic capacity, and antimicrobial stewardship programs (ASPs) may be insufficiently developed to mount effective countermeasures [14,15]. As a result, optimizing empirical antibiotic use in CAP represents both an individual-level and a system-level priority.

In recognition of evolving epidemiological patterns and local antimicrobial susceptibility profiles, the Lebanese Society of Infectious Diseases and Clinical Microbiology (LSIDCM) has promulgated evidence-based national guidelines for CAP management, incorporating risk stratification frameworks and empirical therapy recommendations tailored to regional pathogen distributions and resistance trends [16]. These guidelines emphasize early severity assessment using validated scoring systems. They also highlight the importance of considering patient-specific risk factors for resistant organisms, and judicious selection of empirical regimens that balance adequate coverage with antimicrobial stewardship principles.

Existing literature has established associations between various clinical and demographic variables and CAP outcomes in diverse international contexts; however, regional variations in healthcare delivery systems, pathogen epidemiology, resistance patterns, and population characteristics necessitate context-specific investigations. Moreover, while prior research has examined antibiotic prescribing patterns in Lebanese emergency departments [17], comprehensive analyses focusing specifically on hospitalized CAP patients− a population characterized by greater disease severity, higher comorbidity burden, and more complex clinical decision-making requirements− remain limited. Understanding the determinants of clinical outcomes in this high-risk population is essential for identifying opportunities to optimize care delivery and improve patient outcomes.

Therefore, this study seeks to investigate the clinical outcomes of hospitalized CAP patients in Lebanon and to identify key factors associated with clinical deterioration. In particular, the study evaluates the impact of guideline-adherent empirical antibiotic prescribing on these outcomes. By elucidating the associations between treatment practices and patient trajectories, the findings aim to inform stewardship efforts and reinforce the importance of evidence-based clinical decision-making in CAP management.

## 2. Methods

### 2.1. Study design and setting

This cross-sectional study was conducted using patient data retrieved from five tertiary university hospitals distributed across Lebanon. The participating institutions included two centers in Beirut and one in each of the South, North, and Mount Lebanon regions, ensuring geographical representation.

### 2.2. Study population

The study sample comprised adult patients (≥18 years old) of both genders who had been admitted for CAP and had complete medical records available for review. Exclusion criteria encompassed patients with pneumonia acquired in healthcare or hospital settings (healthcare-associated, hospital-acquired, and ventilator-associated pneumonia), outpatients, patients with multiple concurrent infections, and individuals falling outside the scope of national CAP treatment guidelines, including children and pregnant women. In addition, patients with confirmed or suspected COVID-19 were excluded, as these cases are not covered within the LSIDCM national recommendations for empirical CAP management.

### 2.3. Data collection and variables

Data collection spanned from 2/1/2024–29/6/2024. Patients were identified using the ICD-10 code J18.9. For all eligible cases, we extracted a comprehensive datasets that encompassed demographics, comorbidities, and clinical presentations. Additionally, we recorded laboratory findings and microbiological data; antibiotic susceptibility was specifically interpreted in accordance with the Clinical and Laboratory Standards Institute (CLSI) guidelines. Additionally, details on antibiotic therapy; such as drug selection, dosage, route of administration, and duration; were recorded alongside clinical outcomes (improvement, Intensive care unit (ICU) transfer, or death) and length of hospitalization.

Patient outcomes were evaluated based on clinical stability and resource utilization. Clinical improvement was defined as marked reduction in symptoms and the normalization of vital signs, allowing for hospital discharge without the need for ICU intervention. In contrast, clinical deterioration was assigned to cases involving a worsening clinical state, including sepsis or organ failure, that required ICU transfer or led to in-hospital death.

In addition, each empirical antibiotic regimen was evaluated against the Lebanese national CAP guidelines [16] to determine its appropriateness. Only those regimens that met all four criteria (choice, dosage, route, and duration) were classified as fully appropriate. Treatment outcomes were analyzed in relation to prescribing patterns to assess their impact on patient prognosis.

The empirical antibiotic therapy for adult CAP is clearly outlined in the LSIDCM guidelines [16]. For outpatients and non-ICU inpatients, the preferred first-line regimen consists of a β-lactam in combination with a macrolide. This recommendation reflects the high national prevalence of macrolide-resistant *Streptococcus pneumoniae*. Respiratory fluoroquinolones (RFQ) are reserved as second-line options, primarily for patients with intolerance or allergy to β-lactams, in support of national efforts to limit AMR and collateral damage.

For ICU patients, the LSIDCM guidelines recommend risk stratification for *Pseudomonas aeruginosa*. Individuals are considered at risk if two or more factors are present, such as recent hospitalization, frequent antibiotic exposure, or severe chronic lung disease. ICU patients without these risk factors should receive a non-antipseudomonal β-lactam plus a macrolide or RFQ. Conversely, those identified as at risk for *Pseudomonas* require an antipseudomonal β-lactam combined with either a fluoroquinolone (ciprofloxacin or levofloxacin) or an aminoglycoside plus a macrolide to ensure adequate coverage.

### 2.4. Sample size

Epi Info software was utilized to calculate the target sample size for this study. Applying a 95% confidence level and a 5% margin of error, a minimum of 384 patients was necessary to achieve statistical precision. Initially, 500 patient records were screened (100 per hospital), of which 120 were excluded due to ineligibility (including pediatric patients, pregnant women, and cases with non-CAP diagnoses or multiple infections). A final total of 380 patients met the inclusion criteria and were analyzed.

### 2.5. Statistical analysis

Statistical analysis was performed using IBM SPSS® version 27. Descriptive statistics were used to summarize patient characteristics: categorical variables were reported as counts and percentages, and continuous variables as means with standard deviations (SD). Normality of distribution was assessed using histogram inspection and skewness / kurtosis values.

Bivariate analyses were performed to compare patient characteristics across outcome groups, utilizing Pearson's chi-square or Fisher's exact tests for categorical variables. For continuous data, independent samples t-tests or Mann-Whitney U tests were applied based on the normality of the distribution. Factors potentially associated with clinical outcomes (improvement vs. deterioration/mortality) were then analyzed using a multivariable logistic regression model. This model accounted for several key categories of covariates, including patient demographics, comorbidities, clinical presentation of CAP, and treatment-specific factors such as the appropriateness of the empirical antibiotic regimen. Variable selection was guided by the disjunctive cause criterion to ensure inclusion of potential confounders [18]. Backward stepwise elimination was employed, retaining only variables with p ≤ 0.05 in the final models.

Associations were quantified using adjusted odds ratios (aORs) with 95% confidence intervals (CIs). Statistical significance was set at p-values less than 0.05.

#### Ethical considerations.

The study was approved by the Institutional Review Board (IRB) at Beirut Arab University (IRB code: 2023-H-0092-P-R-0547), on 29/9/2023, along with the ethics committees of all participating hospitals. Patient confidentiality was strictly maintained throughout the study. All patient identifiers were removed by the institution prior to, during, and after data collection, ensuring that the authors had no access to any information that could identify individual participants at any stage. The study was conducted using fully de-identified records in accordance with recognized ethical standards.

#### Informed consent statement.

Informed consent was not obtained for this study because the hospital ethical committees provided de-identified and anonymized data prior to the initiation of data collection to ensure strict protection of patient confidentiality. Given that the study was retrospective in nature, involved no direct patient contact or intervention, and posed minimal risk to participants, obtaining individual consent would have required re-identifying patients, thereby breaching the confidentiality measures established to protect them. In accordance with recognized ethical standards for minimal-risk retrospective research, the IRB and hospital ethics committees granted a waiver of informed consent.

## 3. Results

### 3.1. Patient demographics, clinical severity, and physician profiles

The study included 380 hospitalized patients with a mean age of 67.98 ± 17.01 years, with an almost equal distribution between genders (49.5% male vs. 50.5% female). A significant majority were married (77.6%) and possessed medical insurance coverage (92.1%). Regarding clinical presentation, dyspnea (75.5%) and cough (63.7%) were the most prevalent symptoms, and nearly half of the cohort (49.7%) met the criteria for Systemic Inflammatory Response Syndrome (SIRS) upon admission. The most frequent comorbidities were hypertension (55%), diabetes mellitus (36.3%), and dyslipidemia (26.3%), while 20.3% of patients had pre-existing chronic obstructive pulmonary disease (COPD). According to the CURB-65 severity scale, the majority of patients were classified as moderate to high risk, with 68.9% scoring 2 and 31.1% scoring 3 or higher. Institutional data revealed that most patients were admitted to internal medicine departments (67.9%), while 32.1% required initial ICU admission. Physician demographics showed a predominance of male practitioners (84.7%), with nearly half of the cases (47.4%) managed by pulmonary specialists. The mean length of hospital stay was 7.52 ± 4.02 days. Laboratory findings on admission showed a mean white blood cell count (WBC) of 12.21 ± 4.56 × 10^3^ cells/µL, a mean blood urea nitrogen (BUN) of 30.14 ± 18.09 mg/dL, and an elevated C-reactive protein of 118.65 ± 38.94 mg/L. While the baseline characteristics provide a profile of the overall cohort, significant differences emerged when these variables were stratified by clinical outcome. Table 1 presents a comparative analysis of demographic, clinical, and institutional factors between patients who achieved clinical improvement and those who experienced clinical deterioration.

**Table 1:** Comparative analysis of baseline characteristics stratified by clinical outcome (Total = 380).

Variable	Clinical improvement (Total = 336) n (%) or mean ± SD	Clinical deterioration (Total = 44) n (%) or mean ± SD	N	p-value
**Hospital Area**				
Beirut	143 (88.8%)	18 (11.2%)	161	0.745
North Lebanon	95 (90.5%)	10 (9.5%)	105	
South Lebanon	33 (84.6%)	6 (15.4%)	39	
Mount Lebanon	65 (86.7%)	10 (13.3%)	75	
**Hospital Department**				
Internal medicine	237 (91.9%)	21 (8.1%)	258	0.002*
Intensive care unit	99 (81.1%)	23 (18.9%)	122	
**Physician gender**				
Male	287 (89.1%)	35 (10.9%)	322	0.309
Female	49 (84.5%)	9 (15.5%)	58	
**Physician position**				
Specialist	174 (89.7%)	20 (10.3%)	194	0.674
Attending	113 (87.6%)	16 (12.4%)	129	
Resident	48 (84.2%)	9 (15.8%)	57	
**Physician specialty**				
Pulmonary	160 (88.9%)	20 (11.1%)	180	0.231
Infectious diseases	75 (91.5%)	7 (8.5%)	82	
Internal medicine	72 (82.8%)	15 (17.2%)	87	
Others (Emergency medicine, Cardiology)	29 (93.5%)	2 (6.5%)	31	
**Patients Data**				
Age (years)	67.59 ± 16.86	70.98 ± 18.06	-----	0.215
Length of hospital stay (days)	7.30 ± 3.76	9.18 ± 5.33	------	0.028*
**Gender**				
Male	161 (85.6%)	27 (14.4%)	188	0.093
Female	175 (91.1%)	17 (8.9%)	192	
**Medical coverage**				
No	24 (80%)	6 (20%)	30	0.133
Yes	312 (89.1%)	38 (10.9%)	350	
**Smoking**				
No	229 (89.1%)	28 (10.9%)	257	0.547
Yes	107 (87%)	16 (13%)	123	
**Diabetes mellitus**				
No	218 (90.1%)	24 (9.9%)	242	0.228
Yes	118 (85.5%)	20 (14.5%)	138	
**Hypertension**				
No	156 (91.2%)	15 (8.8%)	171	0.170
Yes	180 (86.1%)	29 (13.9%)	209	
**Dyslipidemia**				
No	244 (87.1%)	36 (12.9%)	280	0.159
Yes	92 (92%)	8 (8%)	100	
**Coronary artery disease**				
No	279 (89.7%)	32 (10.3%)	311	0.116
Yes	57 (82.6%)	12 (17.4%)	69	
**Congestive heart failure**				
No	301 (89.6%)	35 (10.4%)	336	0.061
Yes	35 (79.5%)	9 (20.5%)	44	
**Cerebrovascular disease**				
No	325 (89%)	40 (11%)	365	0.089
Yes	11 (73.3%)	4 (26.7%)	15	
**CNS diseases**				
No	296 (88.9%)	37 (11.1%)	333	0.493
Yes	40 (85.1%)	7 (14.9%)	47	
**Chronic Obstructive Pulmonary Disease**				
No	268 (88.4%)	35 (11.6%)	303	0.954
Yes	68 (88.3%)	9 (11.7%)	77	
**Neoplastic disease**				
No	322 (89%)	40 (11%)	362	0.331
Yes	14 (77.8%)	4 (22.2%)	18	
**Renal impairment**				
No	290 (89.8%)	33 (10.2%)	323	0.048*
Yes	46 (80.7%)	11 (19.3%)	57	
**Allergy**				
No	331 (88.3%)	44 (11.7%)	375	0.643
Yes	5 (100%)	0 (0%)	5	
**Antibiotic use within the past 3 months**				
No	311 (88.1%)	42 (11.9%)	353	0.562
Yes	25 (92.6%)	2 (7.4%)	27	
**Living in a long-term care facility**				
No	310 (88.3%)	41 (11.7%)	351	1.00
Yes	26 (89.7%)	3 (10.3%)	29	
**Clinical Symptoms and Physical Examination**				
**Confusion**				
No	228 (91.2%)	22 (8.8%)	250	0.019*
Yes	108 (83.1%)	22 (16.9%)	130	
**Cough**				
No	120 (87%)	18 (13%)	138	0.500
Yes	216 (89.3%)	26 (10.7%)	242	
**Dyspnea**				
No	75 (80.6%)	18 (19.4%)	93	0.007*
Yes	261 (90.9%)	26 (9.1%)	287	
**Fever**				
No	149 (89.2%)	18 (10.8%)	167	0.666
Yes	187 (87.8%)	26 (12.2%)	213	
**Pleuritic Chest Pain**				
No	270 (88.2%)	36 (11.8%)	306	0.818
Yes	66 (89.2%)	8 (10.8%)	74	
**Decreased Breath Sounds**				
No	186 (85.7%)	31 (14.3%)	217	0.153
Yes	150 (92%)	13 (8%)	163	
**Wheezing**				
No	163 (84%)	31 (16%)	194	0.018*
Yes	173 (93%)	13 (7%)	186	
**Rales/Crackles**				
No	190 (86.8%)	29 (13.2%)	219	0.087
Yes	146 (90.7%)	15 (9.3%)	161	
**Tachypnea**				
No	219 (86.9%)	33 (13.1%)	252	0.285
Yes	117 (91.4%)	11 (8.6%)	128	
**CURB−65 Score**				
2	243 (92.7%)	19 (7.3%)	262	0.001*
3	78 (80.4%)	19 (19.6%)	97	
4	14 (70%)	6 (30%)	20	
5	1 (100%)	0 (0%)	1	
**Empirical Antibiotic Class**				
β-lactam + macrolide	86 (93.5%)	6 (6.5%)	92	0.024*
β-lactam + RFQ	141 (89.2%)	17 (10.8%)	158	
RFQ alone	34 (79.1%)	9 (20.9%)	43	
Antipseudomonal β-lactam + RFQ	60 (92.3%)	5 (7.7%)	65	
Antipseudomonal β-lactam + RFQ +vancomycin	12 (66.7%)	6 (33.3%)	18	
Antipseudomonal β-lactam + macrolide +AG	3 (75%)	1 (25%)	4	
**Carbapenems Included**				
No	300 (90.9%)	30 (9.1%)	330	< 0.001*
Yes	36 (72%)	14 (28%)	50	
**Overall appropriateness of empirical antibiotic therapy**				
Appropriate	143 (96.6%)	5 (3.4%)	148	< 0.001*
Inappropriate	193 (83.2%)	39 (16.8%)	232	
**Appropriateness of antibiotic selection**				
Appropriate	148 (96.7%)	5 (3.3%)	153	< 0.001*
Inappropriate	188 (82.8%)	39 (17.2%)	227	
**Appropriateness of antibiotic dosage**				
Appropriate	283 (90.4%)	30 (9.6%)	313	0.009*
Inappropriate	53 (79.1%)	14 (20.9%)	67	
**Appropriateness of antibiotic route of administration**				
Appropriate	329 (88.4%)	43 (11.6%)	372	0.761
Inappropriate	7 (87.5%)	1 (12.5%)	8	
**Appropriateness of antibiotic duration**				
Appropriate	311 (88.9%)	39 (11.1%)	350	0.632
Inappropriate	25 (83.3%)	5 (16.7%)	30	
**Vital Signs and Laboratory Findings**				
Temperature (°C)	38.0 ± 1.03	38.04 ± 1.08	-----	0.780
Respiratory rate (breaths/minute)	22.54 ± 5.69	22.31 ± 6.24	-----	0.829
Pulse (beats/minute)	87.27 ± 15.79	86.60 ± 18.09	-----	0.818
SBP (mmHg)	125.93 ± 21.68	119.63 ± 20.74	-----	0.106
DBP (mm Hg)	71.92 ± 12.25	69.37 ± 14.91	-----	0.263
WBC × 10^3^ (cells/ µL)	11.76 ± 4.65	13.94 ± 4.23	-----	0.003*
Neutrophils (%)	75.57 ± 15.08	77.33 ± 18.83	-----	0.575
BUN (mg/dL)	29.48 ± 17.65	35.02 ± 20.64	-----	0.045*
Serum Creatinine (mg/dL)	1.11 ± 0.87	1.24 ± 0.084	-----	0.248
Hematocrit (%)	36.54 ± 6.60	30.77 ± 9.08	-----	<0.001*
O2 saturation (%)	92.69 ± 5.87	89.03 ± 9.15	-----	0.108
**Systemic Inflammatory Response Syndrome**				
No	171 (89.5%)	20 (10.5%)	191	0.497
Yes	165 (87.3%)	24 (12.7%)	189	

*Denotes a significant p-value <0.05

### 3.2. Empirical antibiotic use and influence of prescriber characteristics

The most frequently prescribed empirical regimen was a β-lactam plus RFQ combination (41.6%), followed by β-lactam plus macrolide (24.2%). Guideline-adherent empirical antibiotic therapy, defined as appropriate drug choice, dose, route, and duration, was achieved in only 38.9% of cases [19].

While most prescriptions were aligned with guideline-recommended routes and durations, the main deviation stemmed from antibiotic selection. Notably, RFQ-based combinations and monotherapies were frequently used in non-ICU patients, despite guidelines reserving them for more severe cases or penicillin-allergic individuals. Inappropriate dosing, particularly in renally impaired patients, was also documented, largely involving fluoroquinolones and carbapenems.

**Fig 1:**
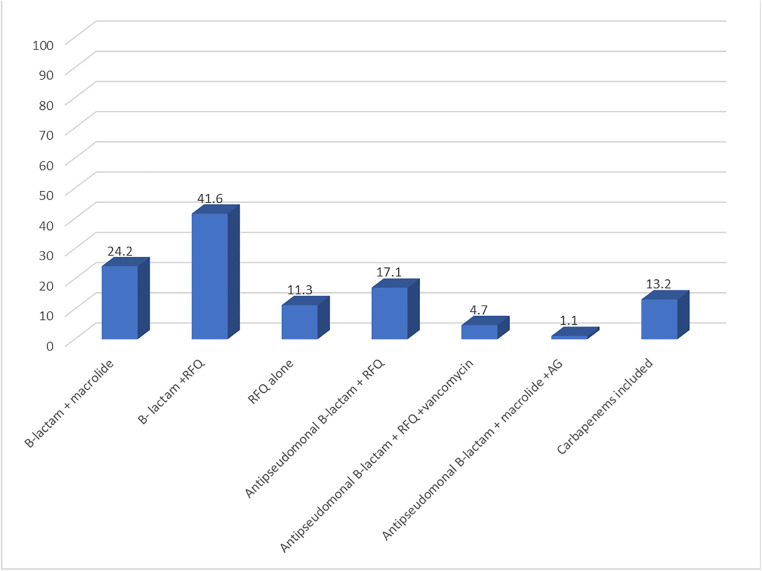
Percentage of empirical antibiotic classes prescribed for hospitalized CAP patients (N=380) RFQ: respiratory fluoroquinolones, AG: Aminoglycoside.

**Fig 2:**
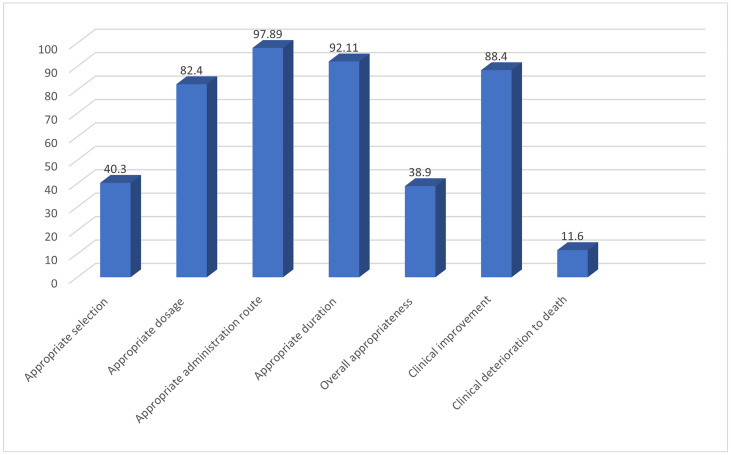
Percentage of appropriate empirical antibiotic prescriptions and clinical outcomes for hospitalized CAP patients (N = 380).

Nevertheless, favorable clinical outcomes were observed in the majority of patients despite the observed deviations from national recommendations. Detailed prescribing patterns and clinical outcomes are illustrated in Figs 1 and 2.

### 3.3. Bivariate analysis of risk factors for clinical outcomes

In this bivariate analysis, demonstrated in Table 1, patients admitted to critical care units had a higher rate of poor outcomes compared to those in internal medicine departments (18.9% vs 8.1%, p = 0.002). Longer hospital stays were observed in the deterioration group (9.18 ± 5.33 vs 7.30 ± 3.76 days, p = 0.028). Renal impairment was significantly associated with worse outcomes (19.3% vs 10.2%, p = 0.048).

Clinical features including confusion (16.9% vs 8.8%, *p* = 0.019), absence of dyspnea (19.4% vs 9.1%, p = 0.007), and absence of wheezing (16% vs 7%, *p* = 0.018) were linked to increased deterioration. Laboratory findings revealed higher white blood cell counts (13.94 ± 4.23 vs 11.76 ± 4.65 × 10³ cells/μL, *p* = 0.003), elevated blood urea nitrogen (35.02 ± 20.64 vs 29.48 ± 17.65 mg/dL, *p* = 0.045), and lower hematocrit (30.77 ± 9.08% vs 36.54 ± 6.60%, p < 0.001) in patients with poor outcomes. Higher CURB−65 scores (particularly scores 3–4) strongly predicted deterioration (*p* = 0.001). Regarding antibiotic therapy, inappropriate selection (17.2% vs 3.3%, *p* < 0.001), dosage (20.9% vs 9.6%, *p* = 0.009), and overall management (16.8% vs 3.4%, *p* < 0.001) were significantly associated with negative outcomes, as was carbapenem inclusion in treatment regimens (28% vs 9.1%, *p* < 0.001).

### 3.4. Factors influencing clinical outcomes: Multivariable logistic regression

Multivariable analysis identified several independent predictors of clinical deterioration. Management by residents (aOR 5.74, *p* = 0.045) or internal medicine specialist (aOR 14.50, *p* < 0.001) was significantly associated with higher odds of clinical deterioration. However, this finding likely reflects differences in patient complexity and case severity rather than provider-related effects. Significant patient factors were advanced age (aOR 1.98), coronary artery disease (aOR 9.95), and congestive heart failure (aOR 10.72). Laboratory markers of risk included elevated WBC count (aOR 1.43) and decreased hematocrit (aOR 0.86).

Disease severity was the strongest predictor, with CURB-65 scores of 3 (aOR 16.28) and 4 (aOR 30.17) markedly increasing risk (*p* < 0.001). Notably, inappropriate antibiotic selection (aOR 18.81, *p* < 0.001) and inappropriate dosing (aOR 5.87, *p* = 0.027) were independently associated with unfavorable outcomes. The Hosmer–Lemeshow test indicated good model calibration (*p* = 0.923), supporting adequate agreement between predicted and observed outcomes. Although the model demonstrated strong predictive performance, the relatively small number of outcome events necessitates cautious interpretation due to potential overfitting (Table 2).

**Table 2:** Multivariable logistic regression analysis of factors influencing poor clinical outcomes in hospitalized CAP patients.

Factor	Wald	Adjusted Odds Ratio(aOR)	95% Confidence Interval	*p*-value
**Physician position (Ref: Specialist)**				
Attending	2.47	1.22	0.93 − 1.48	0.116
Resident	5.41	5.74	1.85 − 18.60	0.045*
**Physician Specialty (Ref: Pulmonary)**				
Infectious diseases	0.68	0.67	0.22 − 1.81	0.433
Internal medicine	10.96	14.50	4.07 − 51.92	<0.001*
**Demographics and Comorbidities**				
Age (years)	8.91	1.98	1.26 − 2.99	0.003*
Congestive heart failure	5.28	10.72	1.42 − 81.02	0.022*
Coronary artery disease	7.66	9.95	2.24 − 43.46	0.006*
**Clinical and Laboratory Data**				
Wheezing	8.91	0.33	0.12–0.91	0.033*
Rales/ crackles	8.06	0.27	0.09 − 0.76	0.014*
WBC × 10³ (cells/µL)	11.83	1.43	1.16 − 1.75	<0.001*
Hematocrit (%)	9.11	0.86	0.78 − 0.96	0.003*
**Severity Indicators**				
CURB-65 Score 3	10.22	16.28	6.32–41.69	<0.001*
CURB-65 Score 4	18.11	30.17	9.67 − 96.34	<0.001*
**Antibiotic Prescriptions**				
Inappropriate antibiotic selection	15.23	18.81	4.03 − 87.35	<0.001*
Inappropriate antibiotic dosing	4.91	5.87	1.94 − 17.82	0.027*

*Denotes a significant p-value <0.05

-Omnibus test of model coefficients p-value < 0.001

-Hosmer and Lemeshow test p-value = 0.923

-Model summary: Nagelkerke R^2^ = 0.723

-Classification table overall percentage = 92.8%

- Constant p-value = 0.323

## 4. Discussion

The analysis of clinical outcomes reflects the consequences of inappropriate antibiotic prescribing. Both bivariate and multivariable analyses demonstrate that inappropriate antibiotic selection significantly increases the risk of poor outcomes (17.2% vs. 3.3%; aOR=18.81), as does inappropriate dosing (20.9% vs. 9.6%; aOR=5.78), demonstrating a strong association between antibiotic stewardship and patient safety. Inappropriate antibiotic selection remains a significant concern, with a study in England reporting that 30.4% of antibiotic prescriptions were inappropriate, particularly in uncomplicated cystitis (62.5%) and bronchitis (48%) [20]. In pediatric patients, 41% of inappropriate prescriptions resulted from improper antibiotic selection, contributing to treatment failures and adverse reactions [21]. Similarly, inappropriate dosing is prevalent, as evidenced by a pediatric study in Bangladesh, where 61.5% of antibiotic doses were incorrect, with overdosing being more common than underdosing [22]. Such dosing inaccuracy has been linked to increased risks of adverse drug reactions and antibiotic resistance, underscoring the necessity of adherence to dosing guidelines [21]. Additionally, inappropriate broad-spectrum antibiotic use in pneumonia cases has been associated with higher readmission rates and prolonged hospital stays [23]. These findings support the implementation of targeted ASPs aimed at improving antibiotic selection and dosing practices within hospital settings.[24]. However, while inappropriate antibiotic use presents significant dangers, the complexity of clinical decision-making and patient variability may justify occasional deviations from standard guidelines.

Our findings revealed several important patient characteristics that influenced clinical outcomes in patients receiving antibiotic therapy. Patients admitted to the ICU experienced significantly poorer outcomes compared to those managed in internal medicine departments (18.9% vs. 8.1%, *p* = 0.002), likely reflecting the greater severity of illness requiring ICU admission. This observation aligns with previous studies demonstrating higher mortality rates in critically ill patients with infections despite aggressive interventions. ICU patients often present with more severe infections, leading to increased mortality rates (20.3% vs. 24.3%) and prolonged hospital stays (median 18 days) compared to non-ICU patients [25]. Moreover, the presence of multidrug-resistant organisms has been correlated with higher mortality and treatment failure, further complicating patient management [26]. Given these risks, timely and appropriate empirical antibiotic therapy is crucial, as studies indicate that inappropriate therapy significantly increases mortality risk [26,27]. Early intervention and tailored treatment strategies based on pathogen profiling can improve clinical outcomes and reduce the burden of antimicrobial resistance [28]. These findings emphasize the critical role of optimized antibiotic management in critically ill patients, reinforcing the need for robust ASPs to enhance patient safety and therapeutic efficacy.

Laboratory and clinical parameters demonstrated significant prognostic value in our patient cohort. Elevated white blood cell counts were associated with poor outcomes, consistent with previous studies showing that pronounced leukocytosis reflects more severe inflammatory responses in pneumonia [29,30]. Similarly, elevated blood urea nitrogen levels suggested impaired renal function or increased protein catabolism, while decreased hematocrit maintained independent predictive value in multivariable analysis, potentially indicating anemia or hemodilution from aggressive fluid resuscitation. These findings align with Waterer et al.‘s observations that hematological parameters can serve as surrogate markers for disease severity [29,30].

Our findings indicate that advanced age, coronary artery disease, congestive heart failure, and pneumonia severity, as reflected by CURB−65 scores, significantly increase the risk of complications in patients hospitalized with CAP. The strong predictive value of CURB−65 scores validates this widely-used tool for risk stratification, comparable to findings by Lim et al. in their original validation study [31].

Similarly, in a previous study, cardiovascular events, including heart failure, atrial fibrillation, and myocardial infarction, are common in severe CAP, occurring in approximately 32% of cases and contributing to increased mortality and prolonged hospital stays [32]. Older adults and patients with preexisting heart disease are particularly vulnerable, as the combination of reduced physiological reserve and severe infection further strains the cardiovascular system, increasing the likelihood of adverse outcomes [33].

Conversely, the presence of dyspnea, wheezing and rales/crackles was associated with better outcomes. This association may reflect earlier recognition and treatment rather than a protective physiological effect. Patients exhibiting these classic symptoms of CAP are rapidly identified as seriously ill and therefore more promptly initiated on escalated therapy, including ICU admission, broad-spectrum antibiotics, and aggressive respiratory support. This aligns with evidence showing that the presence of crackles increases the likelihood of chest radiograph acquisition and early antibiotic administration [34], suggesting that clinicians may be more proactive in diagnosing and managing pneumonia when this clinical sign is present. These findings emphasize the importance of comprehensive risk assessment that incorporates both clinical scoring tools and patient-specific comorbidities to optimize CAP management and improve patient outcomes.

Notably, diabetes and COPD did not remain statistically significant in the bivariate analysis despite their clinical relevance as known risk factors. Patients with these comorbidities are generally prioritized for hospitalization and aggressive therapy, which may mitigate their association with poor outcomes. Prior research found diabetic CAP patients had 3.56 times higher odds of ICU hospitalization [35] and demonstrated greater oxygen support requirements [36]. Similarly, COPD increases the risk of severe CAP, prompting early ICU admission [37]. This may explain why the expected association between certain comorbidities and worse outcomes was attenuated once management interventions were applied.

Finally, physician expertise significantly impacts CAP outcomes, with residents and internal medicine specialists associated with poorer prognoses compared to specialists and pulmonary physicians [38]. This may be due to differences in clinical experience, guideline adherence, and decision-making in managing severe cases. More experienced physicians are better equipped to recognize disease severity and optimize treatment, emphasizing the need for specialized training and adherence to best practices to improve patient outcomes.

The link between inappropriate prescribing and adverse outcomes underscores the critical role of ASPs. In this study, clinical outcomes were primarily driven by adherence to guideline-recommended selection and dosing rather than the antibiotic class itself. These findings suggest that prescribing appropriateness is the primary determinant of patient prognosis in CAP management. While bivariate analysis initially suggested superior improvement with β-lactam regimens, multivariable analysis confirmed that guideline concordance, not the specific drug class, was the true driver of patient outcomes.

Beyond treatment optimization, preventive strategies also play an important role in reducing the economic burden of CAP. One key approach is identifying high-risk patients whose healthcare costs are predicted to be high and targeting these individuals for preventive interventions.. The pneumococcal vaccination remains vital in lessening the severity and complications linked to pneumococcal diseases, especially in the elderly. In Lebanon, the routine vaccination schedule for children involves the 13-valent pneumococcal conjugate vaccine (PCV13). Nonetheless, there is a lack of guidance for adult vaccination in the national immunization schedule [4].

The observed deviations from guideline-recommended therapy highlight the need for structured ASPs to improve guideline adherence and optimize therapy outcomes. Publishing updated national guidelines aids in direct clinical practice, enhances patient outcomes, and constitutes an appropriate starting point in combating the AMR crisis [39,40]. However, our findings suggest that this is insufficient to alter physicians’ clinical practice. Henceforth, the implementation of ASPs is essential for promoting effective and rational antibiotic use. These programs should comprise antibiotic prescribing policies, prospective audit and feedback, education and training, surveillance and reporting, multidisciplinary collaboration, and implementation of guidelines [6,41]. Disease-specific ASPs for CAP that rely on clinical pathways may be particularly high-yield. Various aspects of effective ASPs can enhance CAP management, such as practicing antibiotic de-escalation, shortening the duration of therapy, and adhering to guidelines [42]. These strategies have been previously incorporated into ASPs that have been assessed in studies involving CAP patients, with notable findings in enhanced antibiotic use while preserving positive patient outcomes [43].

Despite the significant findings, the study has several limitations. First, the observational design relied on existing medical records that may lack consistent completeness and detail, potentially introduce information bias and limit causal inferences. Nevertheless, essential covariates were available without substantial gaps, providing a reliable foundation for our analysis.

Although the multi-center design enhanced the comprehensiveness of this study, hospitals from the Beqaa region were not represented, and prescribing practices there may differ slightly. Nevertheless, prior investigations [44,45] have consistently reported high levels of inappropriate antibiotic use across Lebanon, including in the Beqaa, supporting the generalizability of our findings and underscoring the need for nationwide interventions. The dichotomous classification of guideline adherence (adherent versus non-adherent) may also oversimplify the complexity of clinical decision-making, as certain individualized and clinically justified modifications could have been misclassified as non-adherence, potentially inflating estimates of inappropriate prescribing. Furthermore, despite adjustment for multiple covariates in the multivariable analysis, residual confounding cannot be excluded. Physician-level attributes beyond basic demographics such as prescribing tendencies, years of practice, specialty training, and participation in continuing education were not captured. Some clinicians may prioritize personal experience or preferred regimens over strict adherence to guidelines. In addition, institutional influences, including hospital policies and resource constraints, may contribute to variability in compliance with recommended practices.

Moreover, the study did not include PCR testing for viral pathogens and excluded patients with suspected or confirmed viral pneumonia, including COVID-19. While aligned with LSIDCM guideline recommendations for bacterial CAP, this may limit generalizability to settings where viral or mixed etiologies are more prevalent.

In spite of these limitations, this study represents the first comprehensive medical review in Lebanon assessing the impact of appropriate empirical antibiotic therapy on clinical outcomes in hospitalized patients with CAP. Given the scarcity of corresponding data at both national and regional levels, it offers novel insights into outcome-related consequences of prescribing practices and establishes a foundation for future research focused on clinical outcomes.

## 5. Conclusions

This study provides evidence that inappropriate empirical antibiotic therapy for CAP is significantly associated with increased clinical deterioration. Despite national guidelines, adherence is suboptimal, with prescribing patterns influenced by physician rank, specialty, and regional factors. Our findings highlight that guideline-concordant prescribing is critical not only for improving patient outcomes but also for mitigating the escalating threat of AMR.

Therefore, these findings support the need for the implementation of robust ASPs. These programs must integrate targeted prescriber education, clinical decision support, and a national surveillance system to monitor resistance and inform evidence-based practice. Future research should investigate systemic drivers of prescribing behavior and assess the efficacy of stewardship interventions, especially in high-risk patient populations, to translate these findings into improved clinical care and public health policy.
