# Correction: Clinical outcomes in hospitalized patients with community-acquired pneumonia: A comprehensive analysis of associated factors

**DOI:** 10.1371/journal.pone.0350590

**Published:** 2026-06-03

**Authors:** The PLOS One Staff

## Notice of Republication

This article was republished on March 5, 2026, to include the abstract. Please download this article again to view the correct version. The originally published, uncorrected article and the republished, corrected articles are provided here for reference. The publisher apologises for this error.

## Supporting information

S1 File Originally published, uncorrected article. (PDF)

S2 File Republished, corrected article. (PDF)
